# Time Series Analysis of Hand-Foot-Mouth Disease Hospitalization in Zhengzhou: Establishment of Forecasting Models Using Climate Variables as Predictors

**DOI:** 10.1371/journal.pone.0087916

**Published:** 2014-01-31

**Authors:** Huifen Feng, Guangcai Duan, Rongguang Zhang, Weidong Zhang

**Affiliations:** 1 Department of Epidemiology, College of Public Health, Zhengzhou University, Zhengzhou, Henan, China; 2 Department of Infectious Diseases, the Fifth Affiliated Hospital of Zhengzhou University, Zhengzhou, Henan, China; University of Hong Kong, Hong Kong

## Abstract

**Background:**

Large-scale outbreaks of hand-foot-mouth disease (HFMD) have occurred frequently and caused neurological sequelae in mainland China since 2008. Prediction of the activity of HFMD epidemics a few weeks ahead is useful in taking preventive measures for efficient HFMD control.

**Methods:**

Samples obtained from children hospitalized with HFMD in Zhengzhou, Henan, China, were examined for the existence of pathogens with reverse-transcriptase polymerase chain reaction (RT-PCR) from 2008 to 2012. Seasonal Autoregressive Integrated Moving Average (SARIMA) models for the weekly number of HFMD, Human enterovirs 71(HEV71) and CoxsackievirusA16 (CoxA16) associated HFMD were developed and validated. Cross correlation between the number of HFMD hospitalizations and climatic variables was computed to identify significant variables to be included as external factors. Time series modeling was carried out using multivariate SARIMA models when there was significant predictor meteorological variable.

**Results:**

2932 samples from the patients hospitalized with HFMD, 748 were detected with HEV71, 527 with CoxA16 and 787 with other enterovirus (other EV) from January 2008 to June 2012. Average atmospheric temperature (T{avg}) lagged at 2 or 3 weeks were identified as significant predictors for the number of HFMD and the pathogens. SARIMA(0,1,0)(1,0,0)_52_ associated with T{avg} at lag 2 (T{avg}-Lag 2) weeks, SARIMA(0,1,2)(1,0,0)_52_ with T{avg}-Lag 2 weeks and SARIMA(0,1,1)(1,1,0)_52_ with T{avg}-Lag 3 weeks were developed and validated for description and predication the weekly number of HFMD, HEV71-associated HFMD, and Cox A16-associated HFMD hospitalizations.

**Conclusion:**

Seasonal pattern of certain HFMD pathogens can be associated by meteorological factors. The SARIMA model including climatic variables could be used as an early and reliable monitoring system to predict annual HFMD epidemics.

## Introduction

Hand-foot-mouth disease (HFMD) is a common infectious illness in young children, particularly those less than 5 years old. Numerous large outbreaks of HFMD have occurred in Eastern and Southeastern Asian countries, including Singapore, Malaysia, Japan, and China since 1997 [1], [2], [3], [4], [5], which have caused death and neurological sequelae, and have become a growing public health threat. HFMD is an acute enterovirus infection. Human enterovirus 71 (HEV71) and Coxsackievirus A16 (CoxA16) are the major causative agents of this disease [6], [7]. HFMD usually resolves spontaneously, but severe complications can arise, particularly when HEV71 is the causative agent [8], [9]. Currently, neither vaccine nor effective drug against HEV71 is available for human use [10], [11]. Thus, epidemiological surveillance of HFMD and its pathogens is important to take the proper and timely public health interventions to prevent its outbreaks. Early warning of HFMD outbreaks could improve the efficiency of control campaigns and help to take prevention actions to delay the epidemic, thus reducing its impact on health system. HFMD morbidity and mortality would be minimized through earlier and efficient public health response.

Many mathematical models have been developed to predict the occurrence of outbreaks using a combined environmental approach. Seasonal Autoregressive Integrated Moving Average (SARIMA) models allow the integration of external factors, such as climatic variables, to increase their predictive power. This approach has been successfully used to predict the evolution of infectious diseases, such as dengue, vibrio cholera, malaria, and deaths due to influenza [12], [13], [14], [15]. Many studies have indicated that meteorological conditions are the most important factors of HFMD outbreaks [16], [17], [18]. A study in China showed that the weekly number of HFMD cases in the 0–14 years age group increase by 1.86% for every 1°C increase in temperature and by 1.42% for every 1% increase in relative humidity [19].

In this study, we proposed to develop SARIMA models using time series analysis of the number of laboratory-confirmed HFMD hospitalizations. The goals of this study were to characterize whether climatic factors are associated with HFMD epidemics among children and whether inclusion of such factors is useful to predict epidemics with higher precision. This predicable model would be used to facilitate efficient HFMD control.

## Materials and Methods

### Ethics Statement

The study was approved by the Life Sciences Institutional Review Board of Zhengzhou University. It was also approved by the Ethics Committee of each participating hospital: the Ethics Committee of the Fifth Affiliated Hospital of Zhengzhou University, the Ethics Committee of the Third Affiliated Hospital of Zhengzhou University, the Ethics Committee of the Sixth People’s Hospital of Zhengzhou and the Ethics Committee of the Zhengzhou Children’s Hospital. Written informed consent was obtained from the parent of every child participant enrolled in this study.

### Study Area

Zhengzhou, the capital of Henan Province, locates in the central of China, is situated at 34°16′–34°58′ north latitude and 112°42′–114°13′ east longitude, and the total area is 7446.2 square kilometers. The total population of the city amounted to 9.1 million by the end of 2012 (data from the Henan Bureau of Statistics). The population density is the second in China. China lies mainly in the north-temperate zone, characterized by a warm climate and distinctive seasons. In terms of temperature, the nation can be sectored from south to north into equatorial, tropical, subtropical, warm-temperate, temperate, and cold-temperate zones. The average temperature and the average annual precipitation vary greatly from place to place. Zhengzhou lies in the north warm-temperate zone, characterized by a warm climate and distinctive seasons, with a draught spring (March-May) and a hot and rainy summer (June-September). The annual average temperature is 14∼14.3°C, the average annual precipitation is 640.9 mm, and the total sunshine is 2400 hours. The number of clinically diagnosed HFMD reported to the Centers Disease Control (CDC, Zhengzhou, China) was highest in 2009(17,792 cases), and lowest in 2008(1778 cases). Most of the cases occurred from March to June. The average annual incidence was from 26.21/100,000 to 260.56/100,000.

### Meteorological Data

Average atmospheric temperature (T{avg}), maximum atmospheric temperature (T{max}), minimum atmospheric temperature (T{min}), relative humidity (RH), duration of sunshine (SS) and vapor pressure (VP) were routinely measured at the Zhengzhou Meteorological Administration. Daily diurnal variation in temperature was calculated by subtracting the maximum and minimum temperature. These data were available for the period from January 2008 to June 2012 without any missing values, and aggregated on a weekly basis which comprised a total of 234 weeks period.

### Hospitalizations Information of Children with HFMD

The patients were identified according to the diagnostic criteria defined by Ministry of Health. Clinical diagnosis HFMD is characterized by oral vesicular exanthema/ulcers plus vesicular lesions on the hands, and/or feet, and/or buttocks. Laboratory-confirmed cases were clinically diagnosed HFMD with enterovirus-positive, and/or HEV71-positive, and/or Cox A16-positive.

The criteria for HFMD hospitalization included one of the following conditions: total duration of fever≥3 days, peak temperature≥38.5°C on examination, toxic and ill in appearance, recurrent vomiting (at least twice), tachycardia (heart rate≥150/min), breathlessness, poor perfusion (cold clammy skin), reduced consciousness (lethargy, drowsiness, coma), limb weakness, meningitis (neck stiffness or positive Kernig's sign), seizures.

All HFMD cases are the sentinel hospital-based clinical and laboratory HFMD surveillance scheme in China. All children with HFMD in this region hospitalized at the Fifth Affiliated Hospital of Zhengzhou University, the Third Affiliated Hospital of Zhengzhou University, the Sixth People’s Hospital of Zhengzhou and the Zhengzhou Children’s Hospital were eligible for participation in this study. These hospitals host the large-scale dedicated HFMD clinics and wards in Zhengzhou. Almost all of hospitalized HFMD cases were treated in these hospitals. Stool specimens were collected from each child hospitalized with clinical diagnosis HFMD. Participation in the study was voluntary and was proposed to all eligible patients until the target sample was reached. Samples not taken or refusal of participation rate was approximately 12%. Pan-enterovirus, HEV71, and Cox A16 were routinely detected in the clinical laboratory of each sentinel hospital. Moreover, the samples from severe cases were sent to the Centers for Disease Control (CDC, Zhengzhou, China) or the Molecular Laboratory of Zhengzhou University for virus identification. The data collection mechanism has been stable over time, and this routinely collected data can be used for analyzing factors affecting the occurrence of HFMD. Weekly and monthly cases of HFMD were obtained from sentinel hospitals for early detection and measurement of magnitude of epidemics, weekly confirmed cases of HFMD from all laboratories were verified for circulating virus. Data from 2932 samples tested with RT-PCR were subjected to statistical analysis from January 2008 to June 2012.

### Laboratory Analysis

Stool specimens were collected from each child enrolled in this study. These samples were transported immediately at 4°C to the clinical laboratory of each sentinel hospital, the Centers for Disease Control (CDC, Zhengzhou, China) or the Molecular Laboratory of Zhengzhou University and then kept at −70°C until for the detection of HEV71, CoxA16 and universal enterovirus (EV) using the QIGEN Viral RNA kit (QIAGEN, Germany) according to the manufacturer’s instructions. Briefly, The OneStep RT-PCR Kit (QIAGEN, Germany) was used for RT-PCR with a 50 µl reaction mixture containing 3 µl of RNA sample, 5 µl 10× buffer, 2.0 µl dNTP mix (25 mM), 1.0 µl enzyme mix, 0.5 µl RNase inhibitor (40 U/µl), 1.0 µl forward primer, and 1.0 µl reverse primer. The reactions were carried out on 7500 fast PCR instrument (Applied Biosystems), with an initial reverse transcription step at 50°C for 45 min, followed by PCR activation at 95°C for 3 min and 35 cycles of amplification (95°C for 30 s, 50°C for 30 s, 65°C for 60 s). A final extension at 65°C for 10 min was performed. PCR products were observed in 2% agarose electrophoresis. **Table 1** shows the nucleotide sequences of the specific primers and Taq Man probes used in this study.

**Table 1:** Nucleotide sequences of the specific primers and Taq Man probes.

	Primer (5′-3′)	Probe (5′-3′)
Pan-EV	Forward: GCAAGTCTGTGGCGGAACC	(FAM)-AATAACAGGAAACACGGACACCCAAAGTA(TAMRA)
	Reverse: TGTCACCATAAGCAGCCATGATA	
HEV71	Forward: GTTCACCTACATGCGCTTTGA	(VIC)-TCTTGCGTGCACACCCACCG(TAMRA)
	Reverse: TGGAGCAATTGTGGGACAAC	
CoxA16	Forward: CCTAAAGACTAATGAGACCACCC	(TEXASRED)-CTTGTGCTTTCCAGTGTCGGTGCA(TAMRA)
	Reverse: CTAAAGGCAGCACACAATTCG	

### Statistical Analysis

Number of hospitalized children with HFMD and the mean values of the meteorological parameters were calculated for intervals of 7 consecutive days, which are maximal coverage of current weather forecast. Description was performed by time series diagrams. Inferential statistics included Spearman rank correlations, partial and cross correlations, univariate and multiple time series analysis. The number of children hospitalized with HFMD, HEV71-associated HFMD and CoxA16-associated HFMD were considered as the dependent variables. The meteorological data and a seasonal component were considered as the independent variables.

With the goal of predicting the number of HFMD hospitalizations and the major enterovirus infection, SARIMA models were developed. SARIMA models (Box and Jenkins models) have the flexibility to control the autocorrelation of time series data. Four steps were undertaken in the modeling of the number of HFMD and the climate variables. First, using the mean range plot to determine whether the time series of the children hospitalized with HFMD and the climate variables is in a stationary or non-stationary condition. If non-stationary, it has to be transformed into a stationary time series by applying an appropriate transformation (logarithmic, square root, inverse transformation or differencing). Since both HFMD and the climate variables exhibited strong seasonal variation and fluctuations in their yearly means, we adjusted for seasonality by first seasonally differencing the series in the analysis. Second, the temporal structure of seasonal and non-seasonal autoregressive parameters (AR)(P,p), moving average parameters (MV)(Q,q) of the series were determined by assessing the analysis of autocorrelation function (ACF) and partial autocorrelation function (PACF). Once the model was specified, parameters of the model were estimated by using the maximum likelihood method. Third, the goodness-of-fit of the models were determined for appropriate modeling, using the Ljung-Box test measures both ACF and PACF of the residuals, and checking the normality of the residuals. The significance of the parameters should be statistically different from zero. The normalized Bayesian Information Criteria (BIC) and stationary R square (R^2^) were also conducted to compare the goodness-of-fit among SARIMA models. The lowest BIC and the highest stationary R^2^ values was considered good model. Finally, the models developed were verified by dividing the data file into two data sets: the data from the 1^st^ calendar week of 2008 to the 52^nd^ calendar week of 2011 (estimation period) were used to construct a SARIMA model and those between the 1^st^ calendar week to the 26^th^ calendar week of 2012 (evaluation period) were used to validate the model.

We further evaluated whether alternative SARIMA models incorporating climate variables as external regressors have greater predictive power. To facilitate selection of climate variables to be used as external regressors, we computed the association between the number of HFMD hospitalizations and meteorological parameters using spearman rank correlations. Pearson’s or Spearman’s rank correlation was used to further test any correlation among the meteorological parameters. Cross-correlation analysis was used to assess associations between HFMD cases and covariates over a range of time lags (a time lag was defined as the time span between climatic observation and the incidence of HFMD). The time lags chosen for the final model were outcomes of the cross-correlation analysis. To overcome the autocorrelation within each individual series, the correlation coefficients between the number HFMD and climate variables were computed after pre-whitening. Pre-whitening was performed by modeling each time series individually using the SARIMA model. Climatic variables significantly associated to the number of HFMD cases were tested as predictors in multivariate SARIMA model. Similar to the univariate SARIMA model, we estimate the coefficients of multivariate SARIMA associated with the lagged climate variable. The comparison of the SARIMA with and without climatic variables was conducted. The predictive validity of the models was evaluated by calculating the root mean square error (RMSE), which measures the amount by which the fitted values differ from the observed values. The smaller the RMSE, the better the model is for forecasting.

All statistical tests were 2-tailed, and *P* value <0.05 were considered to be statistically significant in terms of an explorative data analysis. For statistical analysis we used SPSS software, version 19 (SPSS).

## Results

### Classification of Pathogens in the Patients with HFMD

Of the 3380 subjects admitted to the isolation wards for treatment between January 2008 and June 2012, 48 were excluded from the protocol analysis for failing to meet inclusion criteria with respect definition of HFMD. 3332 hospitalized with HFMD cases, 2932 children provided stool samples for testing, 201 were severe and 5 died of HFMD. 93.5% patients were under 5 years old, the youngest was 5 months old and the oldest was 12.5 years old. In 2062(69.18%) of the 2932 stool samples tested for HFMD from January 2008 to June 2012, at least one kind of HFMD pathogen was detected. HEV71 (748[36.28%], CoxA16 (527[25.56%]) and other EV (787[38.17%]), were the most common pathogens detected in these samples.

**Figure 1:**
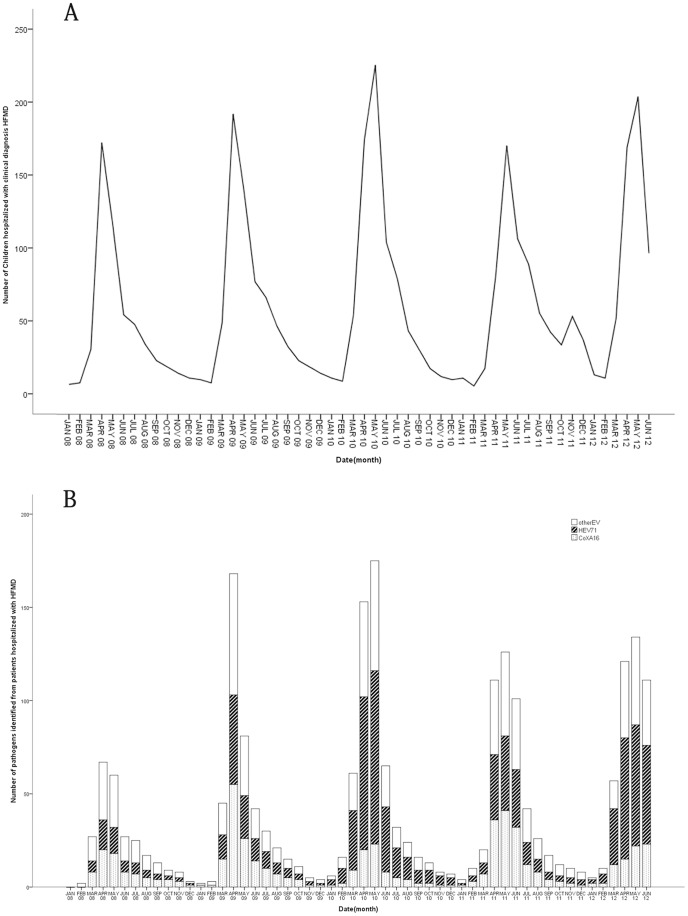
The number of clinical diagnosis cases and the pathogens hospitalized with hand-foot-mouth disease (HFMD) in Zhengzhou, China from 2008 to 2012. The 3 most frequent pathogens leading to hospitalized children with HFMD in Zhengzhou from 2008 to 2012 were, in order, other enterovirus (other EV), Human enterovirus 71 (HEV71) and CoxsackievirusA16 (CoxA16).

The number of clinical diagnosis HFMD cases (**Figures 1A**) and the classification of the pathogens (**Figures 1B**) were shown in Figure 1. HEV71, CoxA16, and other EV were detected all year round, whereas HEV71, CoxA16 and other EV, showed distinctive spring and early summer peaks (**Figures 1**).

### Bivariate Analysis

T{avg}, T{max}, T{min}, RH, SS and VP were significantly correlated with the overall number of HFMD hospitalizations. HEV71 was most strongly correlated with T{avg}, then the CoxA16. We found statistically significant but weaker correlations for the association between RH, SS and these 2 pathogens **(** **Table 2** **)**.

**Table 2:** Spearman rank correlation coefficients for the associations between meteorological parameters and hospitalizations of children with HFMD.

Parameters	HFMD		other EV		HEV71		CoxA16	
	*r_s_*	*P*	*r_s_*	*P*	*r_s_*	*P*	*r_s_*	*P*
VP	−0.654	0.000	−0.619	0.000	−0.553	0.000	−0.561	0.000
T{avg}	0.647	0.000	0.611	0.000	0.533	0.000	0.531	0.000
T{max}	0.627	0.000	0.595	0.000	0.517	0.000	0.523	0.000
T{min}	0.622	0.000	0.579	0.000	0.510	0.000	0.496	0.000
RH	−0.137	0.025	0.125	0.033	−0.172	0.022	−0.151	0.0392
SS	0.235	0.002	0.229	0.002	0.177	0.007	0.272	0.001

Because different meteorological parameters may also be correlated with each other, we analyzed the relationship among these parameters. In fact, average atmospheric temperature was inversely correlated with vapor pressure (*r_s_* = −0.930; *P*< 0.001), but correlated with duration of sunshine (*r_s_* = 0.178; *P* = 0.006), relative humidity (*r_s_* = 0.259; *P*< 0.001).

**Figure 2:**
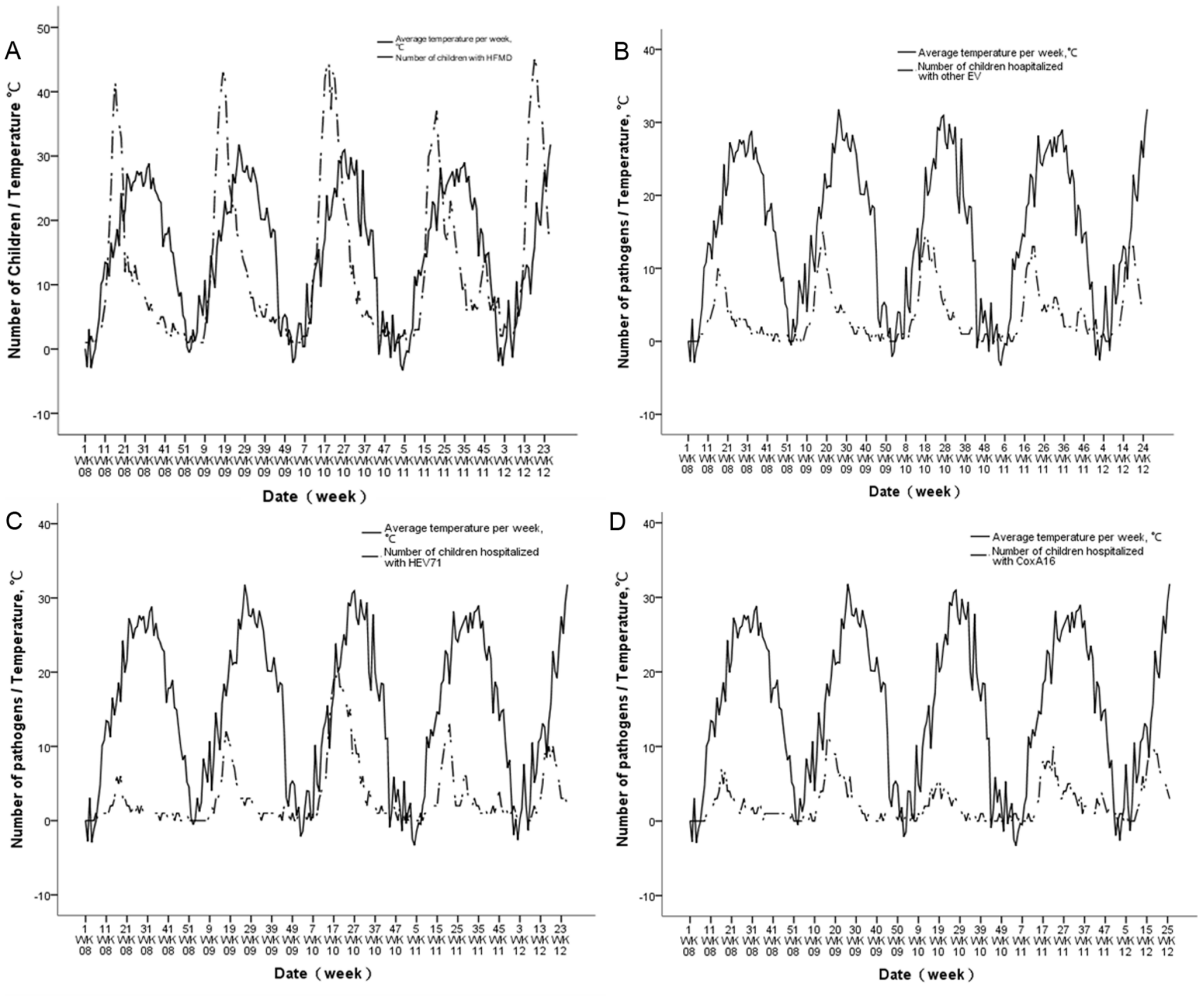
Weekly numbers of hospitalized children with HFMD in Zhengzhou, China from January 2008 to June 2012 compared to crude meteorological variables for the same period. An alternate course is seen between temperature and the pathogens. HFMD (A), other EV(B), HEV71(C) and CoxA16(D).

Accounting for these intercorrelations, associations between meteorological factors and the number of HFMD hospitalization were then analyzed using partial correlations: detection of any of the pathogens was associated with average atmospheric temperatures **(** **Table 3** **)**.The figures also demonstrated temperature and hospitalization caused by the most common pathogens detected over time, showing association of increased activity of HFMD with atmospheric temperatures **(** **Figure 2** **)**.

**Table 3:** Partial correlations between meteorological parameters and hospitalizations of children with HFMD for the adjustment of correlations among other meteorological parameters.

Parameters	HFMD		other EV		HEV71		CoxA16	
	*r_s_*	*P*	*r_s_*	*P*	*r_s_*	*P*	*r_s_*	*P*
VP	−0.048	0.468	−0.052	0.431	−0.011	0.873	−0.091	0.172
T{avg}	0.442	0.001	0.418	0.000	0.349	0.000	0.374	0.000
RH	−0.115	0.085	−0.096	0.153	−0.107	0.109	−0.075	0.259
SS	0.015	0.827	0.017	0.795	−0.032	0.634	−0.075	0.258

### Multiple Analysis

**Figure 3:**
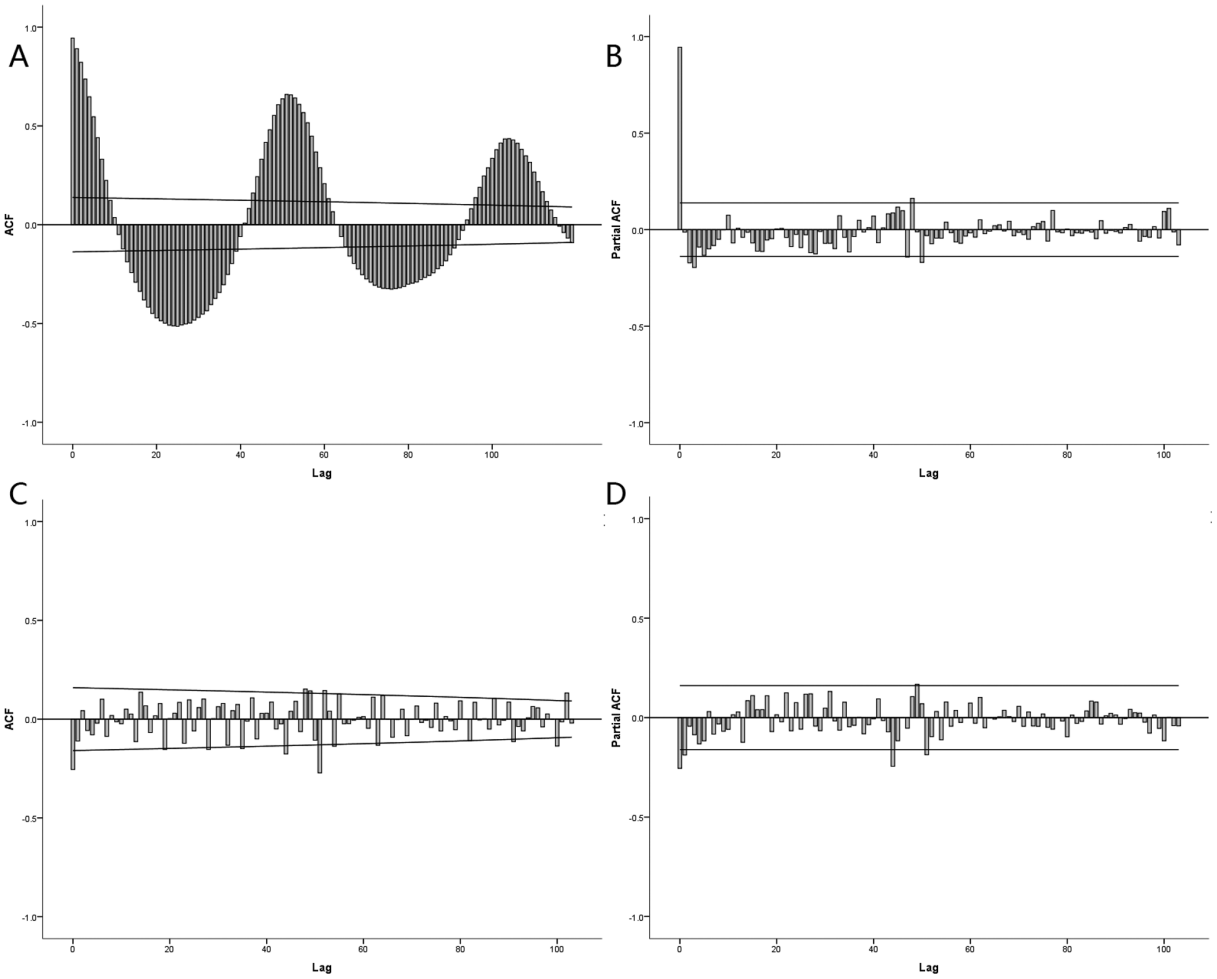
Autocorrelation function (ACF) and Partial ACF (PACF) plot of original and integrated the number of HFMD hospitalizations. A and B) shows ACF and PACF plot of original HFMD hospitalizations. C and D) ACF and PACF plot of integrated HFMD hospitalizations.

**Figure 4:**
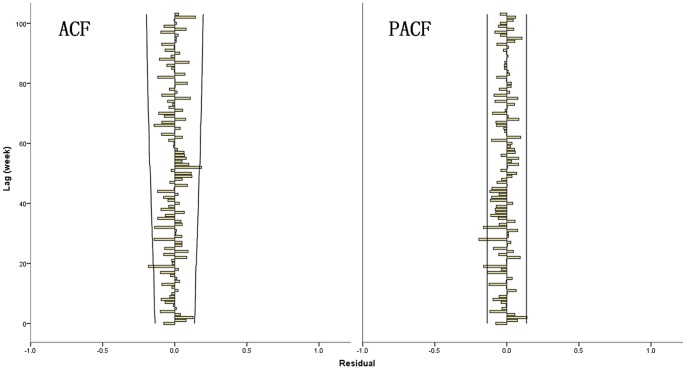
Autocorrelation function (ACF) and Partial ACF (PACF) plot of residuals after applying a SARIMA (1, 1, 1) (1, 0, 0)_52_ model. The x-axis gives the number of lags in weeks and, the y-axis, the value of the correlation coefficient comprised between −1 and 1. Dotted lines indicate 95% confidence interval.

In the first step of the HFMD time series analysis, a square root transformation was performed to stabilize the variance of the series. Then we calculated one time regular differencing for the variable to ensure the time series stationary. The plots of auto correlation function (ACF) and partial auto correlation function (PACF) (**Figures 3A and 3B**) showed the temporal dependence of the number of cases hospitalized with HFMD and confirmed the need to use a SARIMA model with seasonal (P, D, Q) and non-seasonal (p, d, q) parameters. Upon checking ACF and PACF, after differencing, a significant cut offs at one week lag and another at lag 52 weeks were observed on the plot ACF (**Figure 3C**). These two cut offs were less marked on the plot PACF (**Figure 3D**) and evolve more gradually over the time, compared to the plot ACF. The analysis from the correlograms of the series suggests that *p* value should be equal to 1 or 2 and *q* value equal to 0 or 1 of moving average parameters. We fitted the data with several univariate SARIMA (p,d,q)(P,D,Q)s with different orders and excluded the models in which the residual is not likely to be white noise. Among these models, the univariate SARIMA (1,1,1)(1,0,0)_52_ model had both lowest BIC and highest R^2^ values and appeared the best to fit the cases hospitalized with HFMD **(** **Table 4** **)**. The analyses of residuals on ACF and PACF plots assessed the absence of persistent temporal correlation **(** **Figure 4** **)**. The Ljung-Box test confirmed that the residuals of time series were statistically not dependent **(** **Table 4** **)**. The selected SARIMA model fitted observed data from 2008 to 2011. Furthermore, the model was used to forecast the number of HFMD hospitalizations between January and June 2012, and was then validated by the actual observations. The validation analyses indicate that the model had reasonable accuracy over the predictive period (RMSE = 0.377). **Table 4** also described the characteristics of the SARIMA models for the number of HEV71-associated HFMD and Cox A16-associated HFMD hospitalizations.

**Table 4:** Characteristics of SARIMA models for the number of cases hospitalized with HFMD, HEV71-associated HFMD, Cox A16-associated HFMD.

Variables	SARIMA model	AR	MA1	MA2	SAR1	*R* ^2^	BIC	*P*	RMSE
HFMD	(1,1,1)(1,0,0)_52_	0.754	0.623	–	0.375	0.198	2.905	0.339	0.377
HEV71	(0,1,2)(1,0,0)_52_	–	–	−0.234	0.291	0.162	0.529	0.177	1.269
Cox A 16	(0,1,1)(1,1,0)_52_	–	0.563	–	−0.551	0.417	0.488	0.329	1.236

SARIMA: Seasonal Autoregressive Integrated Moving Average model, AR: autoregressive, MA: moving average, SAR: seasonal autoregressive, *R* ^2^: Stationary R-squared, BIC: Bayesian information criteria, *P*: Ljung-Box test, RMSE: Root Mean Square Error.

To include climatic variables (time series) as external variables in the univariate model, a SARIMA model was applied to the time series. We first removed trend and seasonal components of each time series through SARIMA modeling. A regular differencing and a seasonal differencing were applied to the atmospheric temperature. Climatic variables identified as the most interconnected to the number of HFMD were accounted one by one, due to their strong interconnection. The results of the cross-correlations show that the number of children hospitalized with HFMD were significantly positively associated with T{avg} (coefficients = 0.296, *P*<0.05), T{max}(coefficients = 0.207, *P*<0.05) at lag 2 weeks. HEV71-associated HFMD positively associated with T{avg}at lag 2 weeks (coefficients = 0.182, *P*<0.05) and T{max}at lag1 week (coefficients = 0.211, *P*<0.05), while CoxA16-associated HFMD only positively associated with T{avg}at lag 3 weeks (coefficients = 0.190, *P*<0.05) and T{max} (coefficients = 0.183, *P*<0.05) at lag 3 weeks.

**Figure 5:**
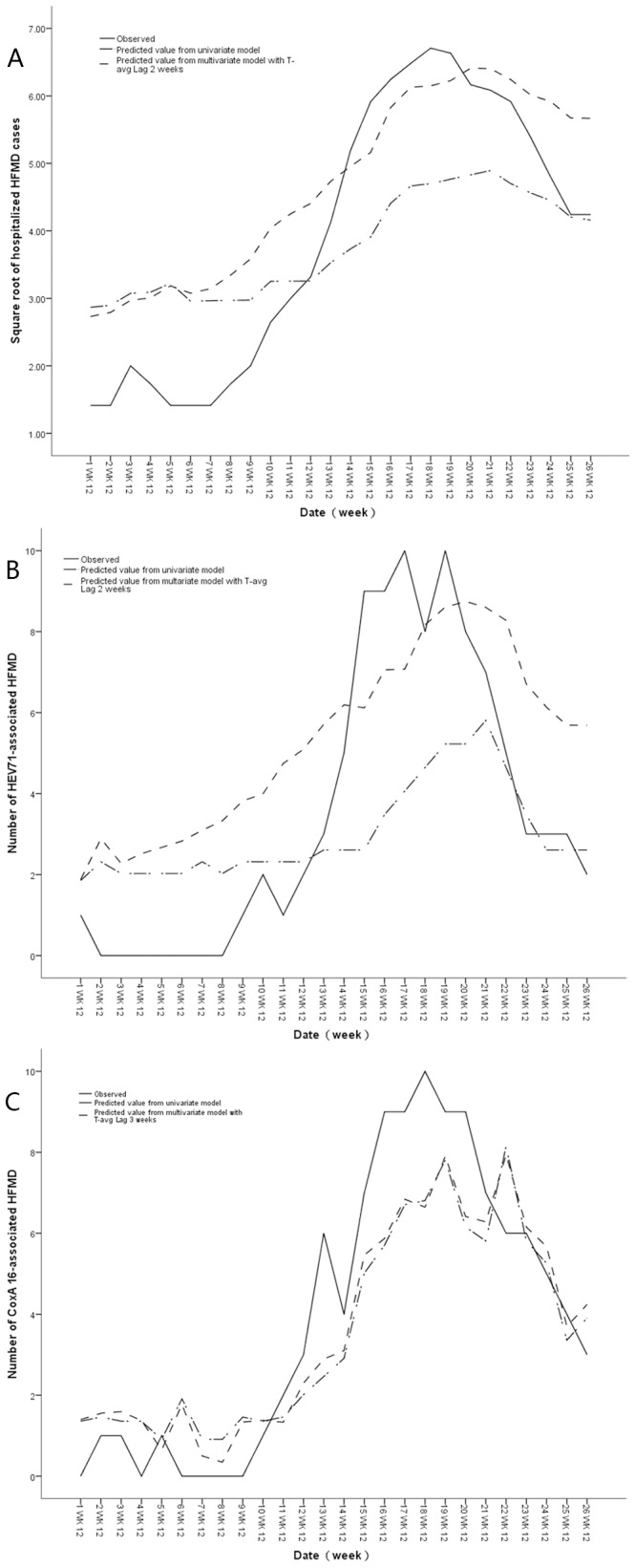
Prediction of square root transformation of the number of HFMD hospitalizations, the number of HEV71-associated and CoxA16-associated HFMD hospitalizations on the basis of a seasonal autoregressive integrated moving average model (SARIMA) model with average atmospheric temperature as the covariate for 2012. Solid line: observed values during the period, dashed line: predicted values for 2012 with and without climatic variables. A: Square root transformation of the number of HFMD hospitalizations, B: the number of HEV71-associated HFMD hospitalizations, C: the number of CoxA16-associated HFMD hospitalizations.

The identification of climate variables that significantly correlated with HFMD hospitalizations were tested with univariate SARIMA models, which were carried out by including external independent variables. Average atmospheric temperature at lag 2 weeks (T{avg}-Lag 2) was the only independent covariate that significantly associated with the number of HFMD hospitalizations in the multiple time series analysis. Overall models the SARIMA (0,1,0)(1,0,0)_52_ associated with T{avg} -Lag 2 weeks is the most appropriate, which has the best fit and highest R^2^. The model estimated with the T{avg} -Lag 2 weeks was a better fit than the model without the variable (Stationary R-squared (Stationary R^2^ ) increased, while the BIC decreased). **(** **Table 4** **,** **Table 5** **)**. The model was used to predict the number of HFMD hospitalizations from January to June 2012, and validated by the actual observations **(** **Figure 5** **)**. The validation analyses indicate that the model increased with the inclusion of T{avg}-Lag 2 weeks (RMSE = 0.352) compared with the model without this variable (RMSE = 0.377).

**Table 5:** Characteristics of multivariate SARIMA models using climate variables for the number of cases hospitalized with HFMD, HEV71-associated HFMD, Cox A16-associated HFMD.

Parameters	HFMD	HEV71	CoxA16	
SARIMA model	(0,1,0)(1,0,0)_52_	(0,1,2)(1,0,0)_52_		(0,1,1)(1,1,0)_52_
MA1	–	–		0.529±0.074
MA2	–	−0.227±0.071		–
SAR1	0.369±0.079	−0.251±0.088		−0.490±0.099
T{avg}-Lag2 weeks	0.019±0.005	0.079±0.026		–
T{avg}-Lag3 weeks	–	–		0.091±0.037
*R^2^*	0.229	0.232		0.402
*BIC*	1.871	0.543		0.627
*P*	0.356	0.585		0.664
*RMSE*	0.352	1.230		1.297

SARIMA: Seasonal Autoregressive Integrated Moving Average model, AR: autoregressive, MA: moving average, SAR: seasonal autoregressive. β: Coefficient, SE: Standard Error, *R* ^2^: Stationary R-squared, BIC: Bayesian information criteria, P*: Ljung-Box test, RMSE: Root Mean Square Error, T{avg}-Lag2 weeks: average atmospheric temperature at lag 2 weeks, T{avg}-Lag3 weeks: average atmopheric temperature at lag 3 weeks.

Multiple time series analysis was also performed for the climate variables on the number of hospitalizations due to HEV71 and Cox A 16 infections. T{avg}-Lag 2 weeks and T{avg} at lag 3 (T{avg}-Lag 3 ) weeks were the independent covariate that significantly associated with the number of HEV71-associated HFMD and the Cox A 16-associated HFMD hospitalizations in the multiple time series analysis, respectively. Models of SARIMA (0,1,2)(1,0,0)_52_,SARIMA (0,1,1)(1,1,0)_52_ shows the fitted models of HEV71-associated HFMD with T{avg}-Lag 2 weeks and Cox A16-associated HFMD with T{avg}-Lag 3 weeks. HEV71-associated HFMD model with T{avg} -Lag 2 weeks was better fit and validity than the univariate model, while the Cox A16-associated HFMD model with T{avg}-Lag 3 weeks didn’t show difference **(** **Table 5** **,** **Figure 5** **)**.

## Discussion

It was observed from this study that HFMD was prevalent year round in this region and peaked between April and July during spring and early summertime. In August, the activity of HFMD fell sharply. However, in 2011 the peak season was in May, one month later than that seen in previous years, followed by a second smaller and unusual epidemic wave of HFMD was observed in middle autumn and winter. Moreover, we also found that the pathogens of HFMD, such as HEV71 and CoxA16, presented a specific annual or biannual specific pattern **(** **Figure 1** **)**. Our findings are in agreement with the incidence of HFMD that has been reported to exhibit seasonal variation in a number of different areas [4], [5]. Epidemiologists have been perplexed by the causes and consequences of seasonal infectious disease for long time, and there is no theory that can alone explain this phenomenon [20], [21]. Environment changes, particularly changes in weather, have been mostly implicated. Annual variation in climate has been proposed to result in annual or more complex peaks in disease incidence, depending on the influence of climatic variables [22], [23]. Many studies suggested that HFMD consultation rates were positively associated with temperature and humidity [16], [17], [18], [19]. Herein, we report that HFMD and the pathogens are significantly associated with meteorological parameters **(** **Figure 2** **)**. This study provided confirmatory evidence for the notion that mean temperature, among various climate variables is the key contributor to HFMD outbreak, which is consistent with results from other studies.

Temperatures and other climatic factors may influence the survival and spread of infectious pathogen in the environment, exposure probability, and the host susceptibility [24], [25], [26]. On the one hand, virus survival under certain climatic conditions could play a role. The survival of the pathogenic organisms outside a host depends on the characteristics of the environment, particularly temperature, humidity, exposure to sunlight, pH and salinity [27], [28].Experimental studies have shown the stability of enteric viruses is influenced by environmental factors such as temperature and relative humidity, which could survive for at least 45 days on nonporous fomites [29]. These findings are supported by epidemiological studies. For example, in the tropics, seasonal peaks in the incidence of enteric viruses have been found to correlate with temperature and relative humidity [16]. This is present study also showed that the activity of HFMD and the pathogens pattern are associated with average atmospheric temperature and the maximum temperature. However, a complicated relationship exists between the micro-environment and enteric viruses, which depends on temperature, salinity and overall levels of water in the environment [28]. It is difficult to predict the incidence of HFMD only on climate since it may peak once or twice a year due to local environment alterations. On the other hand, the probability of transmission of HFMD pathogens might be changed due to host behavior in different seasons. Children are more likely to go outside for playing or swimming during summer than in winter. A lot of previous studies have shown that the summer peaks of polio and other enteric viruses were associated to swimming [30], [31], [32]. Additionally open and weeping skin vesicles, direct contact of contaminated toys and environmental non-hygienic surfaces are other approaches for the spread of enteric viruses infection with the fecal-oral route. In winter time children stay indoor longer, resulting in more contact opportunity and higher transmission among household members. This in turn facilitates transmission of enteric viruses through respiratory droplets. Enterovirus transmitted mainly via faecal-oral, in temperate climates, enteroviral infection occurs primarily in the summer. Therefore, the changes of host behavior, particular patterns of movement and contact, have a potent impact on the seasonality of HFMD.

The time series analysis used in this study produced similar results to previous studies, which made it possible to develop a temporal structure model, especially for seasonal infections. The SARIMA modeling is a useful tool for interpreting and applying surveillance data in disease control and prevention. The model allows the integration of external factors, such as climatic variables, therefore increasing its predictive power [13], [33]. In Japan, HFMD prevalence was positively correlated with the temperature and humidity at lag 0–3 weeks [16]. In Hong Kong, relative humidity, mean temperature, difference in diurnal temperature at 2 weeks’ lag time was positively associated with HFMD consultation rates [17]. And in the city of Guangzhou in China, temperature and relative humidity were significantly associated with HFMD infection with one week lag [19]. We have shown that the increase in average atmospheric temperature was a determining factor in predicting changes of the HFMD incidence. On the contrary, the relative humidity did not appear to play a significant role in this aspect. This study developed a climate-based forecasting model using HFMD hospitalization data collected from 2008 to 2011 in this region, to predict the onset of HFMD of 2012 **(** **Figure 5** **)**. Average atmospheric temperature was identified as a significant predictor for the occurrence of HFMD and the pathogens. After the introduction of the average atmospheric temperature at lag 2 weeks increased the SARIMA models of HFMD and HEV71’s predictive power, which might be implemented in routine surveillance of HFMD and useful for the evaluation of new intervention strategies introduced into this region. However, including weather parameters the prediction model of Cox A 16 could not accurately predict the actual diseases occurrence. Nevertheless, producing accurate predictions using climate data remains a challenge. This study first analyzes the relationship between the most common known HFMD pathogens in children and different meteorological parameters for 5 years, and develops a model for prediction of the number of HFMD hospitalizations on the basis of weather variables in an SARIMA model. The majority of HFMD cases were clinically diagnosed but only a small proportion were laboratory-confirmed in the earlier studies. An early warning of HFMD outbreaks could improve the efficiency of control campaigns and help to take preventive measures. In addition, it provides insight into the local etiology of HFMD, and is helpful in designing preventive strategies. Such early interventions could delay the epidemic, thus reducing its impact on health. Health facilities could adjust their response in terms of availability of beds and mobilization of human and material resources. HFMD morbidity and mortality would be minimized through earlier and proper public health response.

The limitation of this study is that we failed to detect other serotypes of enterovirus except HEV71 and CoxA16 and monitor pathogens in outpatients with HFMD. There is potential to develop an early warning system for HFMD in this region using a predictive model, which would give public health authorities sufficient time to prepare medical equipments and staff in the event of an outbreak. Prediction of outbreaks is imperative in order to develop efficient and cost-effective prevention strategies for HFMD control. However, more work is needed to refine such a model before it is ready for routine use.
